# You Only Find What You Look for: Anchor Bias During the COVID-19 Pandemic

**DOI:** 10.7759/cureus.15416

**Published:** 2021-06-03

**Authors:** Mohammed A Abu-Rumaileh, Nada M Alsharif, Mohammad Abdulelah, Samuel Mueting, Husam Bader

**Affiliations:** 1 Department of Internal Medicine, University of Jordan, Amman, JOR; 2 Department of Internal Medicine, University of New Mexico, Albuquerque, USA; 3 Department of Internal Medicine, Presbyterian Medical Center, Albuquerque, USA

**Keywords:** cognitive bias, covid-19, hypersensitivity pneumonitis, anchoring bias, missed diagnosis

## Abstract

Hypersensitivity pneumonitis (HP) is an immune-mediated syndrome caused by allergen inhalation. High-resolution computed tomography (HRCT) of HP usually shows diffuse ground-glass opacities, but can show centrilobular ground-glass nodules, areas of air-trapping, or fibrotic changes. The clinical presentation of HP as well as the imaging findings can resemble coronavirus disease 2019 (COVID-19) pneumonia. This resemblance, in the absence of a high level of suspicion for other etiologies, led to anchor bias and delayed diagnosis in the case presented here.

## Introduction

Hypersensitivity pneumonitis (HP) is an allergic reaction of the lung parenchyma to variable inhaled organic dusts [1]. It presents predominantly with cough and dyspnea [2]. Chest radiography may be normal or reveal nodular or reticulonodular ground glass nodules [3]. Pulmonary function tests mostly show a restrictive pattern, and CT scans reveal ground-glass opacities [2]. Detailed history-taking is the mainstay of clinical suspicion of HP as there is no specific confirmatory test [4]. A comprehensive multidisciplinary approach is required for the diagnosis of HP as it is suspected in the setting of a constellation of multiple non-specific findings such as exposure history, chest CT scan findings, and bronchoscopic/histopathological findings [5]. After establishing the diagnosis of HP, corticosteroids are the mainstay of therapy [6].

Coronavirus disease 2019 (COVID-19) is an infectious disease caused by the severe acute respiratory syndrome coronavirus 2 (SARS-CoV-2) virus. It was initially identified in Wuhan, China [7]. Its rapid spread worldwide has caused up to this date over 144 million cases and over three million deaths [8]. COVID-19 is a multi-system disease mostly presenting with respiratory symptoms such as cough, fever, and dyspnea [9]. Imaging generally reveals bilateral consolidation on chest radiography [10] and ground-glass opacities on computed tomography (CT) scan [11].

We report a case of delayed diagnosis in a patient with hypersensitivity pneumonitis as a result of anchoring bias.

## Case presentation

A 60-year-old gentleman with previous medical history only pertinent for pre-diabetes mellitus, hypertension, obesity, and depression presented to the emergency department with worsening cough and shortness of breath of six weeks duration. The patient was previously physically active without any respiratory issues or limitations.

The patient started experiencing shortness of breath and mild productive cough that gradually worsened over six weeks. Initially the patient’s symptoms were manageable, but slowly progressed and became debilitating. At the time of presentation, the patient’s symptoms had become so pronounced that he was only able to walk few feet before feeling exhausted and becoming pre-syncopal. 

By the time the patient presented and was admitted through the emergency department, he had visited urgent care three times and had four different negative COVID-19 tests over the span of five weeks; two of the tests were polymerase chain reaction (PCR) and the other two were rapid antigen tests. Patient also had a negative comprehensive viral respiratory panel which included influenza A, influenza B, adenovirus, parainfluenza, rhinovirus, and respiratory syncytial virus. During each visit to urgent care, the patient was prescribed a different antibiotic to cover for bacterial and atypical pneumonia. Antibiotics included amoxicillin/clavulanic acid, azithromycin, and doxycycline.

On admission, the patient denied any fever, chills, orthopnea, paroxysmal nocturnal dyspnea, recent sick contacts, new pets, environmental allergies, or recent travel. The patient reported no chest pain or lower limb edema. His physical examination was pertinent for mild to moderate respiratory distress, hypoxia requiring 8L/min O2 nasal cannula, and pulmonary rales auscultated bilaterally.

Work up prior to hospitalization consisted of four negative COVID-19 tests, as well as a negative comprehensive respiratory panel. We expanded work up to include further infectious labs including coccidioidomycosis antibodies (patient is a resident of New Mexico), Legionella, Mycoplasma, human immunodeficiency virus (HIV) screening, and procalcitonin level. We also investigated a possible autoimmune-mediated pathology by screening antinuclear antibodies. Admission labs are shown in Table 1 and Table 2.

**Table 1:** Blood Chemistry Studies AST: aspartate aminotransferase, ALT: alanine aminotransferase, ESR: erythrocyte sedimentation rate

	01/21/2021	01/25/2021	01/28/2021
Sodium (136 - 145 mmol/L)	139	140	135
Potassium (3.5 - 5.1 mmol/L)	4.8	4.2	4.6
Chloride (98 - 107 mmol/L)	102	107	105
Bicarbonate (22 - 29 mmol/L)	28	28	24
Anion Gap (4 - 13 mmol/L)	9	6	6
Glucose (74 - 99 mg/dL)	81	82	149
Blood Urea Nitrogen (8 - 23 mmol/L)	13	11	27
Creatinine (0.67 - 1.17 mg/dL)	1.03	0.86	1.21
Glomerular filtration rate (mL/min/1.73 sq meter)	79	94	
Calcium (8.8 - 10.2 mmol/L)	9.1	8.5	9.5
Phosphorus (3.4 - 4.5 mg/dl)		3.5	
Total Protein (6.4 - 8.3 g/dL)	7.3	6.7	
Albumin (4.0 - 4.9 g/dL)	3.4	2.7	
Globulin	3.9	4.0	
Bilirubin (Total) (<=1.2 mg/dL)	0.6	0.4	
Alkaline Phosphatase (40 - 129 U/L)	75	67	
AST (0 - 40 U/L)	19	20	
ALT (0 - 41 U/L)	25	20	
Magnesium (1.7 - 2.2 mg/dL)		2.1	
Troponin I (<0.04 ng/mL)		0.017	
Lipase (24 - 151 U/L)		65	
Lactic Acid (0.5 - 2.2 mmol/L)		1.5	
Procalcitonin (<0.15 ng/mL)		0.09	
ESR (1-13 mm/hr)			30
D-Dimer (<0.5 µg/ml)		327	

**Table 2:** Complete Blood Count WBC: white blood cells, RBC: red blood cells, MCV: mean corpuscular volume, MCHC: mean corpuscular hemoglobin concentration, RDW: red cell distribution width

	01/21/2021	01/25/2021	01/28/2021
WBC (4.5 - 11.0 × 10^9^/L)	13.8	13.8	11.5
RBC (4.35 - 5.65 × 10^6^/L)	6.03	5.37	5.50
Hemoglobin (13.5 - 18 g/dL)	18.0	15.7	16.7
Hematocrit (41 - 50%)	55	48	50
MCV (80-100 fL)	91	90	92
MCHC (33.4 - 35.5 g/dL)	33	32.6	33.2
RDW (%)	14.1	13.9	13.5
Platelets (150 to 400 × 10^9^/L)	256	259	245
Neutrophils (%)	77	76	77
Lymphocytes (%)	8	9	10
Monocytes (%)	9	9	10
Eisonophils (%)	5	6	3
Basophils (%)	1	0	0
Absolute Lymphocytes (1.0 - 4.8 × 10^9^/L)	1.2	1.2	1.1
Absolute Neutrophils (1.8 - 7.8 × 10^9^/L)	10.8	10.5	8.9

Chest CT scan showed diffuse bilateral ground-glass opacities that were symmetric in appearance predominantly involving the upper lung fields with no significant septal thickening seen or focal consolidations. Echocardiogram was normal.

**Figure 1:**
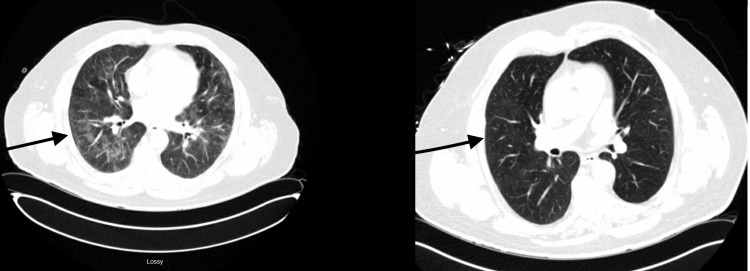
CT Chest Admission CT chest (left) shows bilateral alveolar infiltrates felt to represent viral pneumonia. Six weeks follow-up CT chest (right) shows significant improvement in the previously described bilateral alveolar infiltrates.

Upon further history-taking with emphasis on potential exposures, the patient recalled that he had helped a friend clean up a house that was purchased for renovation. The house was over 50 years old and had an attic that was apparently not cleaned for years, notably heavily covered in dust and dead insects. The patient’s symptoms started a few days after cleaning up the attic, but he did not think the two events were related at the time. Given the timeframe of the exposure, the progression of symptoms, and the negative results of extensive infectious and rheumatological workup, the diagnosis of hypersensitivity pneumonitis was entertained. A bronchoscopy was discussed with the patient, but he opted to postpone unless his symptoms worsened. A trial of steroids was initiated with high-dose prednisone. The patient’s symptoms and oxygen requirements significantly improved after two days of starting prednisone. The patient felt almost “back to normal” within three days. The patient was discharged on steroids and Pneumocystis jirovecii pneumonia (PJP) prophylaxis with outpatient follow up. He was titrated off steroids slowly. A repeat CT chest six weeks after completion of steroids showed significant improvement of the bilateral opacities as shown in Figure 1.

## Discussion

Medical errors result in more than 250,000 deaths yearly with iatrogenic causes ranking third [12]. Misdiagnosis occurs in nearly 15% of cases in medical practice, with around 43% of diagnostic errors being related to clinical assessment [13].

Cognitive biases are believed to be one of the major contributors to misdiagnosis, yielding close to 75% of diagnostic errors in internal medicine practice. Cognitive bias can result from relying on clinical impressions more than a systemized analytical process in reaching the final diagnosis [14,15]. Anchoring bias is a common and extensively studied type of cognitive bias, best described as sticking to one’s initial impression and adjusting related findings to reach a presumed diagnosis. Per a study published by Tversky and colleagues, anchoring bias is reportedly highly prevalent in clinical practice and isn’t restricted to laymen [16,17].

Clinical bias is a complex problem that occurs frequently in everyday practice. Clinical biases are carried out subconsciously, which makes overcoming this problem even more challenging. Several studies affirm that clinicians diagnose pathologies that are similar to the "classical" pattern of presentation of similar cases [18]. This bias tendency understandably becomes more pronounced when evaluating a patient with symptoms overlapping those of an ongoing pandemic.

In our case, the patient presented with respiratory complaints amid a pandemic of respiratory illness. Multiple physicians who evaluated the patient were fixated on COVID-19 pneumonia despite objective data consistently not being suggestive of the pathology and treatment not being effective. This case serves as an example of how strongly our medical judgement is influenced by previous similar cases. It also accentuates the magnitude anchor bias can have in clinical decision making.

This case also sheds light on the cornerstones for patient care: history and physical examination. Despite the shift toward investigation-centered practice in the last decades, history-taking remains the main contributor to reach the correct diagnosis. It is estimated that thorough history-taking and physical examination alone contribute 76% towards reaching the diagnosis [19,20]. We encourage physicians to revert back to the basics and consider a thorough rather than focused history and physical examination in cases where the diagnosis is in question or the patient is not improving.

## Conclusions

Anchoring bias is a common and well-studied bias. The frequency of anchoring bias likely increases when a larger number of patients’ presentations become more uniform, such as during a pandemic. Thorough, rather than focused, history and physical examination can be helpful when a diagnosis is in question.
